# Numerical Analysis of Concrete Hydration Heat Impact on Frozen Soil Temperature around Cast-in-Place Piles

**DOI:** 10.3390/ma17174375

**Published:** 2024-09-04

**Authors:** Yueyue Wang, Xuesong Mao, Qian Wu, Peichen Cai, Min Ye, Shunde Yin

**Affiliations:** 1 School of Highway, Chang’an University, Xi’an 710064, China; yywangnuli@163.com (Y.W.); wuqian@chd.edu.cn (Q.W.); peichencai@chd.edu.cn (P.C.); 2 Department of Civil and Environmental Engineering, University of Waterloo, Waterloo, ON N2L 3G1, Canada; shunde.yin@uwaterloo.ca; 3 School of Construction Machinery, Chang’an University, Xi’an 710064, China; mingye@chd.edu.cn

**Keywords:** permafrost, cast-in-place piles, hydration heat, construction seasons, boundary condition, thermal disturbance

## Abstract

The hydration heat generated during the concreting of cast-in-place piles causes thermal disturbance to the surrounding permafrost, leading to its thawing. This further affects the stability of the pile foundation and degrades the construction progress. To explore the influence mechanisms of the concrete hydration heat on the permafrost temperature field around the pile, as well as that of different construction seasons on the pile-side boundary conditions and permafrost temperature field, monitoring results of on-site tests and numerical simulation were used to analyze the distribution law of the pile soil temperature field in space and time, and the pile-side boundary conditions and permafrost temperature field during construction seasons. The results show that the temperature trend of the pile foundation can be divided into three stages: a rapid rise phase (0∼2 d), a rapid decline phase (2∼10 d), and a slow decline and stabilization phase (10∼90 d). As the radial distance from the pile center decreases, there occur a corresponding acceleration in temperature increase and an elevated maximum temperature rise (MTR). The influence range of the molding temperature and the hydration heat is about 1∼2 times the pile diameter and less than 1.5 m in the depth direction. Compared to the atmospheric temperature, there is a lag in the change in the permafrost temperature caused by accumulation of ground temperature, and the significant difference between the two leads to an increased rate of heat exchange at the boundary condition. Conducting drilling operation and cast-in-place pile construction in the cold seasons is conducive to reducing the thermal disturbance to the permafrost around the pile in permafrost areas.

## 1. Introduction

Permafrost refers to soil or rock that remains at a temperature below 0 °C for at least two consecutive winters and the intervening summer [1,2]. The formation, existence, and evolution of permafrost thermal conditions are primarily controlled by artificial disturbances and climate. Cast-in-place piles are often used for large construction projects, such as for bridge pile foundations in permafrost regions [3,4]. The thermal disturbance of cast-in-place piles on permafrost foundations is significant [5,6]. The heat introduced during the construction process can alter the original dynamic equilibrium state of the foundation [7,8]. This thermal disturbance prompts a rapid increase in the ground temperature surrounding the piles, leading to the thawing of the permafrost within a certain radius, which results in a reduction in the freezing strength of the permafrost and the bearing capacity of the foundation.

When the pile foundation construction has been completed for a period of time, the hydration heat released inside the pile foundation decreases significantly, and with the action of air temperature and ground temperature, the temperature of the cast-in-place piles and the surrounding permafrost gradually drops below zero, resulting in refreezing. The time taken for the soil to refreeze is important, as the thawing of the permafrost affects the form of force exerted on the sides of the concrete piles and the mechanical properties of the pile–soil interface [9,10].

Many scholars have conducted a large number of studies on the temperature variation in the refreezing process of cast-in-place piles in perennial permafrost areas [11,12,13,14]. Li et al. used the finite element method for numerical analysis to investigate the thermal distribution characteristics within the pile–soil interface subsequent to the construction of concrete piles. The study elucidated that thermal increment occurred more rapidly and attained higher values nearer to the pile’s core, with the refreezing process completing from the bottom up [15]. Yu et al. studied the refreezing process of the cast-in-place pile with different pile diameters and found that the refreezing time of the pile foundation with larger cement particle diameter was longer, and the refreezing time of 1.2 m pile diameter was 1.14 times that of 1.0 m pile diameter [16]. The refreezing rate of pile foundation had a strong relationship with the local ground temperature, concrete mold temperature, and the hydration heat [17,18]. Wang et al. concluded that a continuous low-temperature hydration environment can significantly reduce the degree of hydration exothermy at all ages of cement hydration [19]. Chen et al. and Sen et al. investigated the impacts of the concrete curing temperature and the hydration heat release degree on the thermal disturbance within pile foundations using laboratory experiments [13,20], which revealed that while the concrete curing temperature had a more pronounced immediate effect on the temperature variations of the pile foundation, the influence of the heat generated by hydration was more prolonged.

The existing literature primarily focuses on the effects of the molding temperature and hydration heat on the refreezing process of cast-in-place piles and the temperature field of the surrounding soil. However, in permafrost regions, significant variations in the atmospheric temperature inevitably impact the hydration heat release process of cast-in-place piles and the temperature field of the permafrost, which, in turn, affects the bearing capacity of the pile foundation and the construction schedule. Nevertheless, studies on the impact of the atmospheric temperature on the hydration heat release of cast-in-place piles and the temperature field of the surrounding soil under different seasonal conditions remain scarce. This research gap limits a comprehensive understanding of the relationship between the construction season and pile foundation performance. Therefore, this study fills this gap by conducting innovative research on the effects of the construction season on the temperature field around cast-in-place piles and its subsequent impact.

Based on these considerations, the paper uses the No. 0 pile of the Junqu Bridge on Provincial Highway 224 in Qinghai Province as a case study. It integrates data from in situ physical tests and numerical simulations to systematically investigate the change process of the pile and the soil temperature field after pile foundation construction in permafrost regions. Furthermore, this paper separately investigates the effects of the construction seasons on the pile-side boundary conditions, and the impact of the molding temperature of cast-in-place pile on the surrounding permafrost temperature field under different construction seasons (April, August, and October). This study is important for the next step to analyze the temperature field on the strength variation of the pile foundations and subsequent superstructure construction progress.

## 2. On-Site Monitoring Test

### 2.1. Geological Survey

**Figure 1:**
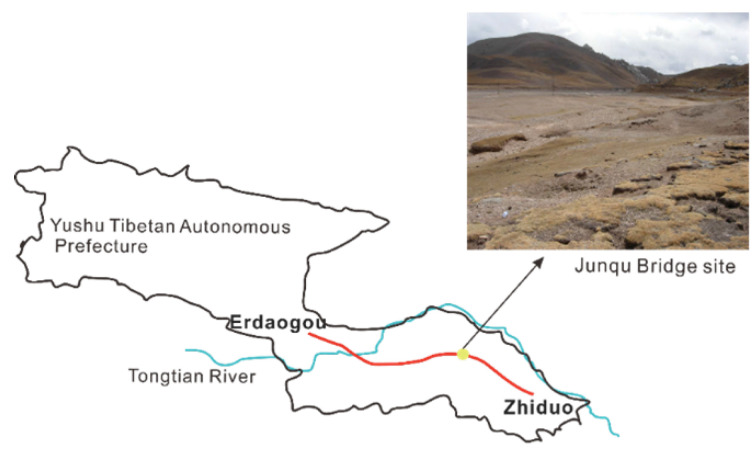
Bridge foundation location.

The test site is located at the Junqu Bridge on a section of Provincial Highway 224 in Qinghai Province (Figure 1), at an altitude of approximately 4615 m, and is classified as an ice-rich permafrost area. The permafrost layer has a thickness ranging from 2.6 to 17.5 m, with the upper limit of permafrost extending from 1.2 to 8.5 m. The area has an average annual temperature of −1.6 °C. The Junqu Bridge is situated 200 m downstream of the Junqu River and spans a wide and shallow riverbed. Based on drilling exploration, the strata in the bridge location primarily consist of Quaternary Holocene alluvial and diluvial pebbles and medium sand underlying Holocene pebbles. The specific characteristics of each stratum are shown in Table 1. Pile No. 0 of the Junqu Bridge, selected as the prototype for the test pile foundation [21], with the design parameters, are presented in Table 2.

### 2.2. Temperature Monitoring Program

The construction of the pile foundation and the burial of the sensors took place in August. Temperature monitoring employed a PT100 temperature sensor, which was composed of probe and cable. The material of the probe was seamless 304 stainless-steel, and the temperature range of the sensor was −50∼200 °C. The temperature sensors were placed in the nanotubes to monitor and collect the internal temperature data of the piles once the concrete was poured. A total of 15 temperature sensors were installed for the experiment, the probes were buried at 0.5 m from the center of the pile, and the sensors were arranged from the top of the pile downwards as follows: one sensor every 1 m within the 0∼10 m range, and one sensor every 2 m within the 10∼20 m range. The temperature sensors inside the pile were connected to an external interface through PVC pipes, and temperature data were subsequently collected at set intervals using a temperature data logger.

## 3. Comsol Computational Model

### 3.1. Mathematical Control Equations

A typical element, with domain *V* and boundary Γ, is assigned to the space x and time systems of reference t, respectively [22]. The response of the elements is controlled by the following system of equations:

(1) $$\nabla^{T}\sigma +\rho c\overset{\cdot }{T}\;=Q_{\infty }\overset{\cdot }{a}\;\;\;\mathrm{in}V$$

(2) ε=∇T inV

(3) σ=−kε inV

(4) $$n^{T}\sigma =\bar{q}\;+h_{N}\left(T-T_{a}\right)\;\;\mathrm{on}\Gamma_{N}$$

(5) $$T=\bar{T}\;+h_{D}n^{T}\sigma \;\;\mathrm{on}\Gamma_{D}$$

In these equations, *k* is the local conductivity matrix; *c* is the specific heat capacity; $\bar{T}$ is the prescribed temperature vector; $T_{a}$ is the ambient temperature; *n* is the normal vector, which indicates the direction of heat flux at the boundary; and $h_{N}$ and $h_{D}$ are the convective heat transfer coefficient at the $\Gamma_{N}$ boundary and thermal conductivity at the $\Gamma_{D}$ boundary, respectively.

The domain equations are completed by the problem’s initial conditions, which can be specified either through prescribed temperature and hydration degree fields, *T* and α, or, to maintain generality, in terms of their time rates:

(6) $$T=T_{0};\;\alpha =\alpha_{0}\;\mathrm{at}\;t=t_{0}\;\mathrm{in}\;V$$

(7) $$\overset{\cdot }{T}\;={\overset{\cdot }{T}\;}_{0};\;\overset{\cdot }{\alpha }\;={\overset{\cdot }{\alpha }\;}_{0}\;\mathrm{at}\;t=t_{0}\;\mathrm{in}\;V$$

In these equations, σ is the heat flow, ρc defines the volumetric specific heat, $Q_{\infty }$ represents the potential heat of the hydration reaction (the total heat that would be released upon full hydration of all cement, means α=1), $\nabla^{T}$ is the transpose of the gradient vector (or its adjoint in non-Cartesian systems), and *k* is the local conductivity matrix.

The model established by the COMSOL Multiphysics was an axisymmetric plane, and calculations were performed using a cylindrical coordinate system (8). The hydration heat of cement was age-dependent (9). This article implements the exothermic process of the hydration heat by defining the hydration heat generation rate (HGEN) [23], and its calculation formula is as follows (10).

(8) $$\left[C+L\rho_{d}w\delta \left(T-T_{f}+\frac{T_{d}}{2},\frac{T_{d}}{2}\right)\right]\frac{\partial T}{\partial t}=\frac{1}{r}\frac{\partial }{\partial r}\left(\lambda r\frac{\partial T}{\partial r}\right)+\frac{\partial }{\partial z}\left(\lambda \frac{\partial T}{\partial z}\right)+Q$$

where *C* is the volume-specific heat capacity of the soil; *L* is the latent heat of the phase change between ice and water, generally taken as 334 kJ/kg; $\rho_{d}$ and *w* represent the dry density and water content (including ice) of soil, respectively. The δ function is the derivative of the 0–1 step function concerning *T* and *T* is the temperature; $T_{f}$ and $T_{d}$ are the upper and lower temperature limits of the phase transition interval, respectively. *t* represents time, λ is the thermal conductivity of the soil and *Q* is the hydration heat; *r* and *z* are the coordinates in the cylindrical coordinate system.

(9) $$Q\left(\tau \right)=Q_{0}\left(1-e^{-m\tau }\right)$$

(10) $$HGEN=\frac{dQ}{d\tau }W_{c}=mQ_{0}e^{-m\tau }W_{c}$$

where $Q\left(\tau \right)$ is the cumulative hydration heat at age τ, in kJ; $Q_{0}$ represents the final hydration heat as it approaches infinity, in kJ, which in this article is taken to be 330 kJ. m is a constant related to the type of cement, the specific surface area, and the casting temperature [24]. When the casting temperature is 5 °C, m = 0.295; at 10 °C, m = 0.318; at 15 °C, m = 0.34; at 20 °C, m = 0.362. $W_{c}$ is the amount of cement per cubic meter of concrete, in kg/m^3^; for this paper, 400 kg/m^3^ is used.

The phase transition of water in permafrost occurs at a certain small range of temperatures [25]; it is assumed that the transition takes place within the temperature range of ($T_{m}\pm \Delta T$). Given the relevant thermodynamic parameters of the material, the sensible heat capacity method can be employed to calculate the phase transition. Taking the effect of the latent heat of phase transition as the volumetric heat capacity, the equivalent heat capacity C(T) and λ(T) expressions (11) and (12) are obtained.

(11) $$C\left(T\right)=\left\{\begin{array}{c} C_{f},T<\left(T_{m}-\Delta T\right)\\ \frac{C_{f}+C_{u}}{2}+\frac{L}{2\Delta T},\left(T_{m}-\Delta T\right)\leq T\leq \left(T_{m}+\Delta T\right)\\ C_{u},\left(T_{m}+\Delta T\right)<T \end{array}\right.$$

(12) $$\lambda \left(T\right)=\left\{\begin{array}{c} \lambda_{f},T<\left(T_{m}-\Delta T\right)\\ \lambda_{f}+\frac{\lambda_{u}-\lambda_{f}}{2\Delta T}\left[T-(T_{m}-\Delta T)\right],\left(T_{m}-\Delta T\right)\leq T\leq \left(T_{m}+\Delta T\right)\\ \lambda_{u},\left(T_{m}+\Delta T\right)<T \end{array}\right.$$

where $\lambda_{f}$ and $\lambda_{u}$ are the thermal conductivities of frozen and thawed soils, respectively; $C_{f}$ and $C_{u}$ are the volumetric heat capacities of frozen and thawed soils; and $T_{m}$ is the freezing temperature.

### 3.2. Computational Model

**Figure 2:**
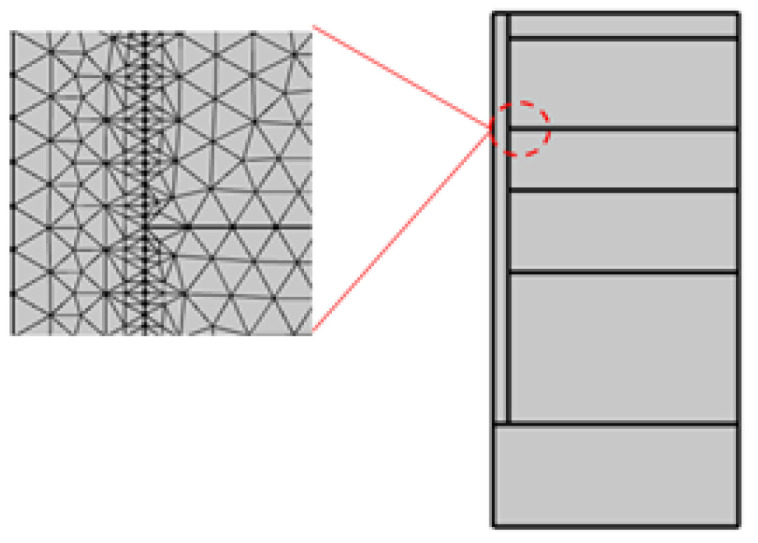
COMSOL computational model.

The No. 0 pile of Junqu Bridge was used as the analysis model. The pile length was 20 m, the pile diameter was 1.5 m; the boundary width × height was 12 m × 25 m. The pile tip of 1.22 m was in the river, and a two-dimensional axisymmetric model was established by using symmetry (Figure 2). To improve the calculation accuracy, the mesh density was chosen to be extremely fine [22]. The values of the thermodynamic parameters and the concrete-related parameters of the calculated soil layers are shown in Table 3.

### 3.3. Boundary Conditions and Initial Values

The heat transfer state on the boundary surface was not considered in the calculation model. Local meteorological data of Qinghai Province were selected for the upper boundary, and the effect of the ground heat flow was considered for the lower boundary, so a heat flux density of 0.03 W/m^3^ was set. The left boundary was the axisymmetric boundary, and the right boundary was the adiabatic boundary.

The initial temperature is as follows:

(13) $${\left.T\left(r,z,t\right)\right|}_{t=0}=T_{1},0\leq r\leq 0.75m$$

(14) $${\left.T\left(r,z,t\right)\right|}_{t=0}=T_{2},0.75<r<12m$$

where $T\left(r,z,t\right)$ is the node temperature of point $\left(r,z\right)$ in the model at any time t; $T_{1}$ is the sum of the concrete entering temperature and the adiabatic temperature rise; $T_{2}$ is the initial ground temperature of permafrost before concrete pouring, and the observed ground temperature in the field is taken for calculation.

## 4. Monitoring Result Analysis

**Figure 3:**
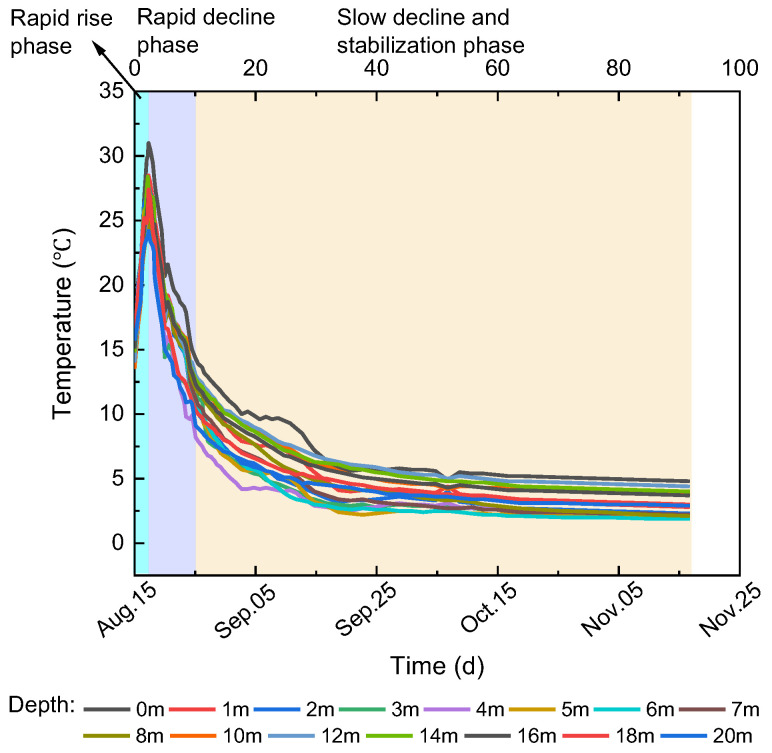
Variation curve of pile-side temperature with time.

The curves of the temperature sensor readings with time at different depths on the pile side of pile 0 are shown in Figure 3. The overall law of sensors at different depths with time was first rising and then falling. Based on the different rates of change, the temperature variation process of the pile foundation can be divided into three stages: I. rapid rise phase (0∼2 d), II. rapid decline phase (2∼10 d), and III. slow decline and stabilization phase (10∼90 d). The main reason for this is the continuous release of hydration heat from the concrete within the pile foundation, with the rate and magnitude of heat release gradually decreasing over time.

In stage I, the rapid release of hydration heat caused the temperature of the pile foundation to rise abruptly. The highest temperature was 31 °C, 16 °C higher than that of the concrete entering the mold, and the average temperature change rate was +5.40 °C/d. In stage II, the cooling rate of the pile foundation was faster—the average temperature change rate was −1.98 °C/d. In stage III, the cooling rate of the pile foundation gradually slowed down and tended to be stable. After 90 days, the internal temperature of the pile foundation dropped to about 2 °C, a decrease of nearly 90%, and the average temperature change rate was −0.1 °C/d.

**Figure 4:**
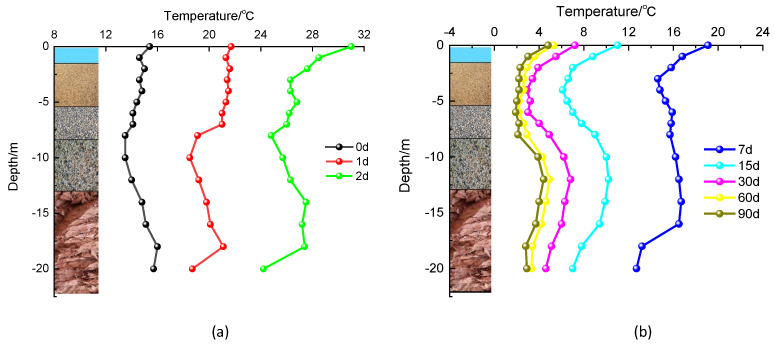
Variation curve of pile-side temperature with depth: (**a**) rapid rise phase; (**b**) rapid decline and slow decline and stabilization phase.

Figure 4 shows the variation curves of the measured temperatures with depth at different ages on the pile side, which had similar “S-shaped” trends. Specifically, when the cast-in-place pile was in the fine sand layer (the depth was less than 5 m), the temperature at the side of the pile decreased gradually with increasing depth; when the cast-in-place pile was in the gravel, boulder, and part of the mudstone layers (the depth was greater than 5 m), the temperature rebounded and rose, following a relatively stable temperature change process; and when the depth exceeded 17.5 m, the temperature of the pile foundation decreased rapidly.

The analysis suggested that the cause was related to the thermal parameters of the soil surrounding the pile and the latent heat of fusion. Wet fine sand, compared to slightly wet gravel, had a higher thermal conductivity, allowing the heat generated by the hydration heat to dissipate more quickly through the surrounding soil; additionally, the fine sand layer had a higher ice content, and the ice crystals in the soil required the absorption of latent heat when melting, causing the temperature rise trend to be slower than in soil layers at other depths. The boulder layer had larger particles and a higher porosity than the gravel layer, and the specific heat capacity and thermal conductivity of the pebble layer were lower. Therefore, the pile foundation temperature had a better heat dissipation effect in the boulder layer than in the fine sand layer, but was slightly worse than in the gravel layer. In addition, the fully weathered mudstone behaved as a clayey soil with a high heat capacity, allowing the pile foundation to dissipate heat faster, and the heat diffusion volume at the pile bottom was larger than that of the pile body, resulting in a faster reduction in the temperature at the pile bottom.

## 5. Simulation Results

### 5.1. Comparison with Test Site Data

According to the results of test site monitoring (M) and COMSOL Multiphysics (C), the temperatures of the pile 0.5 m from the center with ages of 1, 2, 15, 30, and 90 d at different depths were selected, respectively, and the mean absolute error (MAE) of different ages was calculated (as shown in Table 4).

The monitoring results of the test site were basically consistent with the numerical simulation results. The MAE values for the remaining intervals were below 3 °C. This indicated that the numerical model was adept at forecasting the thermal influence of the cast-in-place pile on the surrounding soil. The observed discrepancies in the data may be attributed to several factors as follows: the release rate and quantity of hydration heat were influenced by the concrete’s composition and proportions; the boundary conditions employed in the simulations may not accurately mirror the actual site conditions, such as potential errors in the external environmental temperature; the concrete’s hydration reaction, being a complex chemical process, might not be precisely or thoroughly represented in the numerical model; the finite element simulations typically required simplifications and assumptions regarding the subject matter, including the omission of local temperature gradients and the complexities of boundary conditions. Moreover, the accuracy of the measured data will be affected by the precision of the sensor and the frequency of data acquisition.

### 5.2. Changes in the Range of Effects of Concrete Hydration Heat

**Figure 5:**
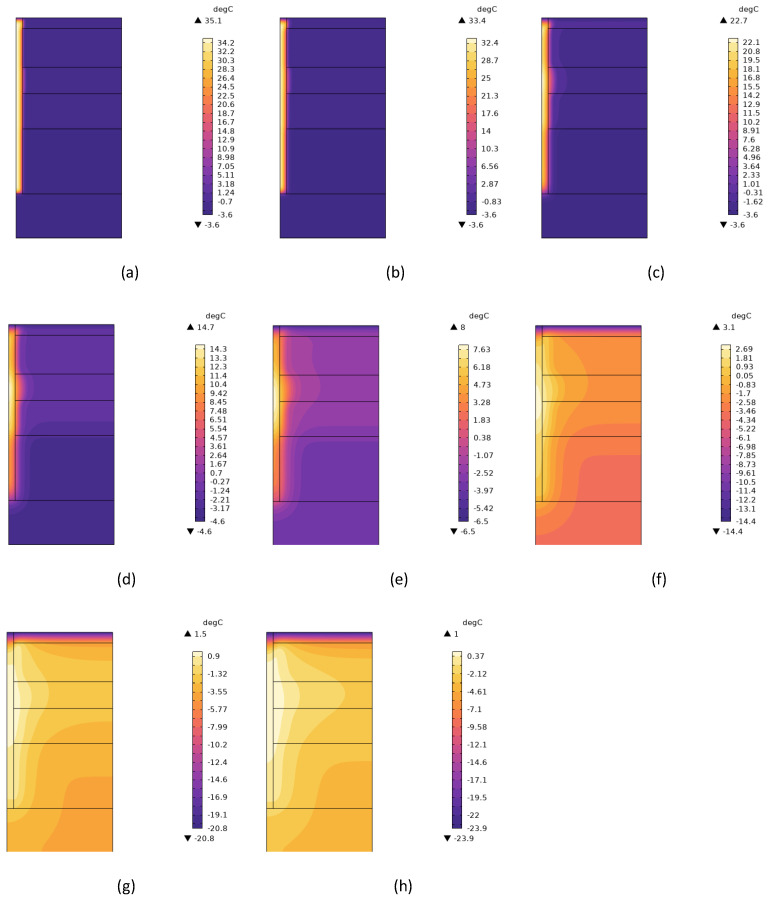
Distribution map of the pile and soil temperature field at different ages: (**a**) 1 d; (**b**) 2 d; (**c**) 7 d; (**d**) 15 d; (**e**) 30 d; (**f**) 60 d; (**g**) 90 d; (**h**) 120 d.

The simulation calculations indicated that the average temperature difference at the pile side was 0.06 °C for the ages of 110 to 120 d, 0.06 °C for 120 to 130 d, and 0.06 °C for 130 to 140 d. They showed that the temperature around the pile had basically stabilized and remained unchanged, indicating that the hydration heat of the concrete no longer affected it. To accelerate the simulation speed and ensure more stable calculations, a computational step length of 120 d was selected for this simulation program. Figure 5 shows the distribution diagram of the temperature field of the pile and soil at 1, 2, 7, 15, 30, 60, 90, and 120 d after concrete pouring when the molding temperature was 15 °C.

Figure 5 shows that after the concrete pile was poured, the effect of the concrete hydration heat led to a rapid increase in the temperature of the pile, and gradually spread outward to affect the temperature of the surrounding soil. With increasing age, the isotherm of soil around the pile showed a parabolic shape along the depth direction. The soil temperature within 4 m below the pile bottom was still affected by the hydration heat. The above phenomenon illustrated that the effect of the concrete hydration heat on the temperature of the pile and the surrounding soil gradually decreased with increasing depth, and decreased as the distance from the pile grew.

### 5.3. The Variation Law of Pile Soil Temperature with Space

**Figure 6:**
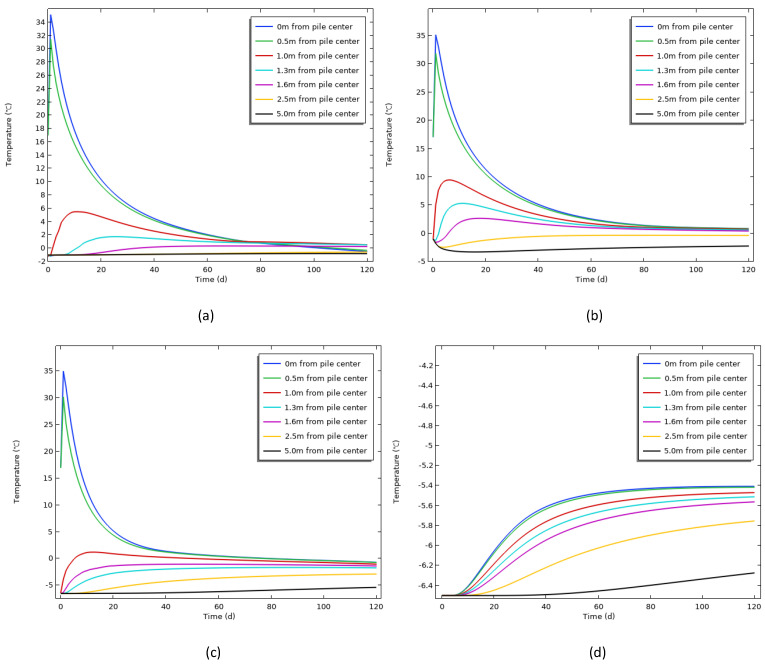
Variation in the pile soil temperature with time in different spaces: (**a**) 2 m depth; (**b**) 8 m depth; (**c**) 14 m depth; (**d**) 22 m depth.

To analyze the variation in the pile and soil temperature with radial distance at different depths, seven characteristic points were selected along the pile diameter direction. These points were located at the pile center, and at radial distances of 0.5 m, 1.0 m, 1.3 m, 1.6 m, 2.5 m, and 5.0 m from the pile center. Additionally, observation points were chosen along the depth at 2 m, 8 m, 14 m, and 22 m to monitor the temperature pattern at these characteristic points changes with age and radial distance (Figure 6).

Figure 6 indicates that within the depth range of 2∼14 m, the temperature of the pile soil varied in a similar pattern over time, with a quicker rise in temperature closer to the pile center and a higher maximum temperature rise (MTR). The temperature at the pile center reached 35 °C of MTR on 1 d. The temperature at 0.5 m from the center of the pile was almost the same as the monitoring value, which indicated that the model was accurate. In particular, within the range of 1.6 m from the pile center, the MTR changed significantly, which was manifested as a hysteretic phenomenon with increase in the distance from the pile center, indicating that the influence of the hydration heat on the soil around the pile gradually spread. After that, with decrease in the hydration heat release rate and release amount, the temperature inside the pile decreased rapidly, and the average temperature change rate was −1.74 °C/d. Compared with the center temperature, the temperature change rate in the surrounding soil along the radial direction gradually slowed down. For example, at 14 m depth, the average temperature change rates at 0.5 m, 1 m, and 1.3 m from the center of the pile were −0.32, −0.07, and −0.01 °C/d, respectively. As the age of the concrete continued to grow, the temperature of the pile and soil began to decrease slowly. After approximately 100 d of construction, the pile and soil temperatures were essentially similar to the natural ground temperature.

Figure 6a shows that the pile soil temperature at the surface was significantly affected by the atmospheric temperature. When the age had exceeded 70 d, by this time it was November, and the local temperature had dropped to −4 °C and continued to cool down. Consequently, the pile soil temperature curve at each location continued to decrease. Since the depth of 22 m was far from the concrete pile (as shown in Figure 6d) and was not affected by the atmospheric environment, the temperature change was only related to the hydration heat and the lower boundary conditions. Therefore, the soil temperature there always remained below 0 °C, and the range of change was within 1 °C. In addition, the permafrost beyond 1.6 m from the center of the pile was basically unaffected by the hydration heat of the concrete, and the temperature change was small. The temperature difference at the depth of 2 m was 2.8 °C at the most, and it was always in the negative temperature state, which indicated that the influence range of the hydration heat was about 1∼2 times the pile diameter.

**Figure 7:**
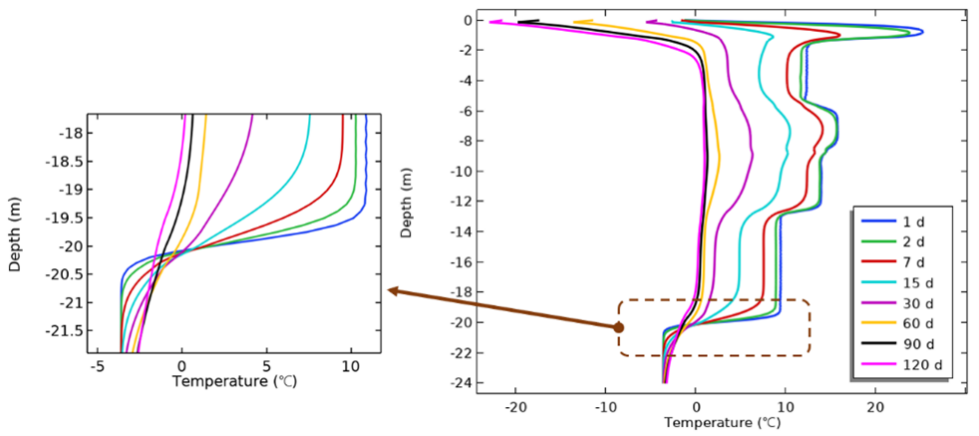
Relationship between pile-side temperature and depth at different ages.

The model results can better demonstrate the temperature change of the actual pile and soil (Figure 6). On this basis, the temperature of the pile–soil contact surface was further analyzed with respect to the depth to study the spatial change trend (Figure 7). At a time 1 d after the concrete was poured, the temperature of the pile side reached its maximum (27.2 °C). The temperature variation of the pile side near the river was larger. With increase in depth, the temperature of the pile side decreased gradually, which indicated that the process of pile refreezing developed from bottom to top. The pile bottom can not only dissipate heat in the horizontal direction, but also in the deeper permafrost, resulting in a very rapid temperature reduction rate at the pile side below the 19 m depth. The temperature difference of permafrost below 21.5 m was basically negligible, so the influence range of hydration heat in the depth direction was less than 1.5 m. With decrease in the hydration heat, the temperature of the pile side gradually reduced with age. When the age was 98 d, the temperature of the pile side at different depths dropped below 0 °C, which proved that the soil around the pile had completed refreezing.

### 5.4. The Influence of Construction Season on Hole Side Soil in the Process of Hole Formation

**Figure 8:**
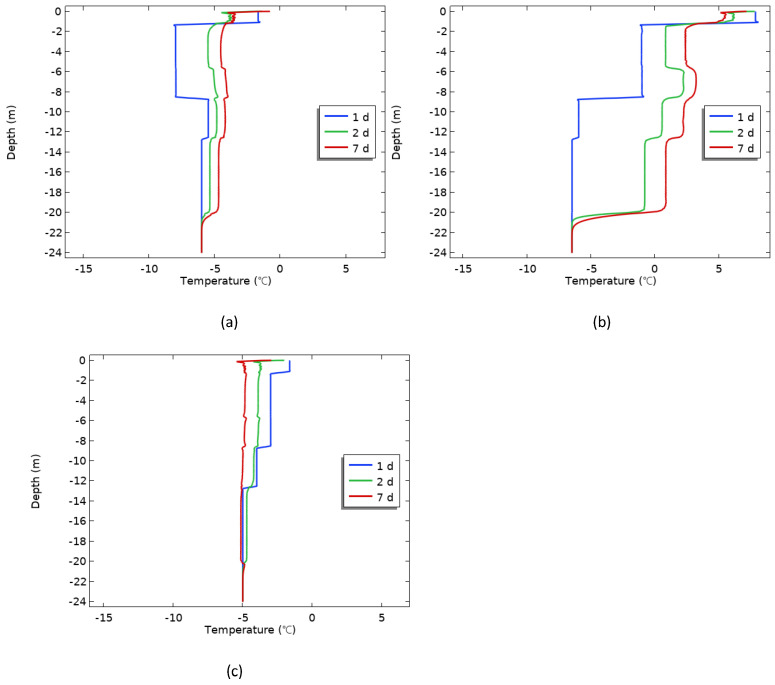
Temperature variation of the boundary condition after hole formation: (**a**) April; (**b**) August; (**c**) October.

The disturbance of soil temperature in construction is inevitable [26], and the operation of drilling and desilting, as well as the direct exposure of the side wall of the foundation pit to the atmospheric environment, can destroy the temperature structure of the permafrost. To clarify the influence law of the pore formation process on the hole side temperature and further study the influence mechanism of the construction season on the hole wall as a boundary condition, the boundary condition of the hole wall was set as convective heat flux, the upper boundary condition and initial ground temperature were set as the actual temperature of the construction season in April, August, and October, respectively [27], and the other boundary conditions remained unchanged. The test results are shown in Figure 8.

Figure 8 shows the temperature variation trend of the boundary condition after the hole formation under different construction seasons. Affected by the heat exchange between the atmospheric temperature and the permafrost temperature, Figure 8c reflects that the soil temperature in the borehole wall gradually decreased within a week, gradually increased in April and August (Figure 8a,b), and the soil temperature of the borehole wall even reached a positive temperature on August.

The low temperatures in April were near 0 °C, while the permafrost had completely frozen through under the influence of the winter conditions, resulting in ground temperatures that were lower than the air temperatures. After the hole was made, the permafrost of the hole’s wall was directly exposed to the atmospheric environment. The significant temperature difference led to the permafrost gradually absorbing external heat, causing the temperature of the hole’s wall to increase progressively. Similarly, the temperature in October was negative, and continued to fall. Meanwhile, due to the absorption of heat by the permafrost layer during the summer, there was an accumulation of ground temperature. Under the influence of lower air temperature, the heat from the permafrost layer was gradually released [28], and the ground temperature began to decrease.

### 5.5. Influence of Molding Temperature on the Temperature Field of Pile Soil under Different Construction Seasons

The ground temperature obviously varies in different construction seasons, and the permafrost temperature is the key to influencing the formation of foundation-bearing capacity and concrete strength, which will directly affect the subsequent processes. The permafrost temperature field is bound to be thermally disturbed to varying degrees by the concrete molding temperature [29]. Therefore, investigating the changes in soil temperature in the short term after construction in different seasons has a certain reference value for the selection of the construction period for bridge pile foundations in permafrost areas. Based on local climate monitoring data over many years, this paper divided the year into four seasons: spring (March, April, and May), summer (June, July, and August), autumn (September, October, and November) and winter (December, January, and February). Considering that reducing the casting temperature of concrete can shorten the construction period of pile foundation in the high-temperature permafrost area [30], and the insulation effect of concrete temperature on the construction site is greatly affected by air temperature, the model sets the casting temperature of 5 °C in April, 10 °C in August, and 6 °C in October in the construction season to conduct simulation tests.

**Figure 9:**
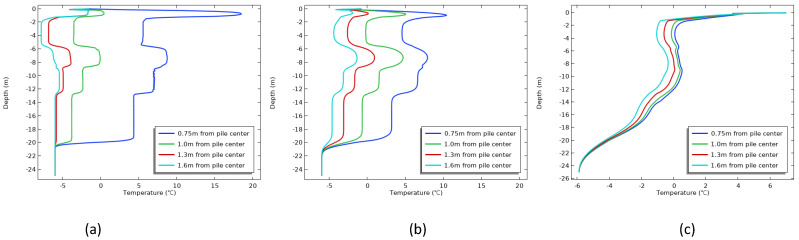
The temperature changes with the depth of each monitoring point at different ages after construction in April: (**a**) 1 d; (**b**) 7 d; (**c**) 80 d.

Figure 9 shows the variation in temperature with depth at different monitoring points under the condition that the construction season is April. When the age was 1 d (Figure 9a), the soil temperature at the pile side grew extremely fast, and the highest temperature was 18.5 °C. The closer the distance from the center of the pile, the more drastic the soil temperature change. When the age reached 7 d (Figure 9b), the soil temperature on the pile side had begun to refreeze, with the highest temperature of 10.4 °C. In contrast, the permafrost temperature within 1.0∼1.6 m from the pile center was in a rising stage. As the age increased, it was summer at 80 d and the soil temperature around the piles was basically below 0 °C (Figure 9c), indicating that the soil around the piles had completed refreezing. As the average value of the ambient temperature in that month was 5∼6 °C, it resulted in a positive river temperature at 0∼2 m depth.

**Figure 10:**
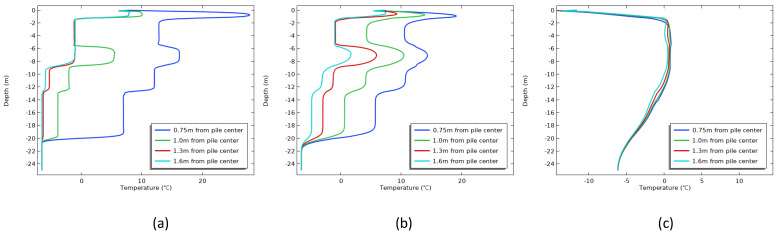
The temperature changes with the depth of each monitoring point at different ages after construction in August: (**a**) 1 d; (**b**) 7 d; (**c**) 107 d.

Figure 10 shows the variation in temperature with depth at different monitoring points under the condition that the construction season is August. When the age was 1 d (Figure 10a), under the influence of the hydration heat, the soil temperature at the pile side rose rapidly, and the highest temperature reached 27.8 °C. With distance from the pile center, the permafrost temperature changed less. As the age increased to 7 d (Figure 10b), the diffusion of heat energy led to a decrease in the permafrost temperature on the pile side, while the permafrost temperature further away gradually increased; in particular, the permafrost temperature at a distance of 1.0 m from the pile center was already above 0 °C and began to melt. In the range of 2∼5 m depth, the soil temperature changed very slowly, on the one hand, because the shallow permafrost temperature in August was higher, which was −1 °C; on the other hand, the fine sand layer had a high ice content, and the ice crystals in the soil needed to absorb a lot of latent heat when melting. When the age was 107 d (Figure 10c), the permafrost around the pile was basically refrozen.

**Figure 11:**
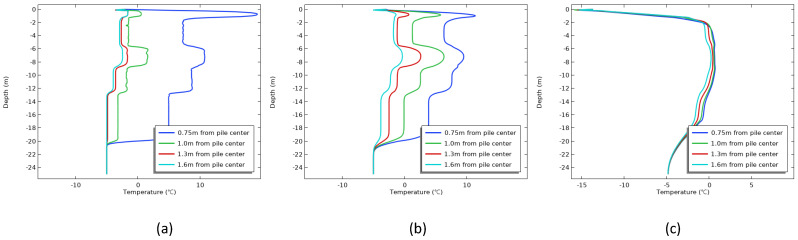
The temperature changes with the depth of each monitoring point at different ages after construction in October: (**a**) 1 d; (**b**) 7 d; (**c**) 95 d.

Similarly, it can be seen from Figure 11 that the soil temperature at the side of the pile rose rapidly one day after the construction of the cast-in-place pile was completed in November (Figure 11a), and the highest temperature reached 19.2 °C. As the distance from the pile center was closer, the permafrost temperature changed sharply. When the age was 7 d (Figure 11b), the variation law of the permafrost temperature field was similar to that in Figure 8b. With increased age, the temperature of the soil around the pile was basically below 0 °C at 95 d (Figure 11c), indicating that the soil around the pile had completed refreezing. At this time, the ground temperature was lowered in the coldest month of January [26,31,32], but the time required for the soil around the pile to refreeze was longer than that of the construction season in April, which further illustrated that compared with the ground temperature in the current month, the choice of construction season was more important in the construction of cast-in-place piles.

The MTR and refreezing time of permafrost 0.75 m, 1.0 m, and 1.3 m away from the pile center were selected at a depth of 8m. The results are shown in Table 5. They indicate that the MTR increase in the permafrost diminished with increasing distance from the pile, the refreezing process concluded earlier, and the residual hydration heat led to the completion of refreezing on the pile side. During construction in April, as compared to August, the MTR at distances of 0.75 m, 1.0 m, and 1.3 m from the pile center exhibited reductions of 7.4, 5.1, and 3.8 °C, respectively, and the refreezing time decreased by 27, 25, and 24 d at these respective distances. Compared to October, the MTR of the soil at 0.75 m, 1.0 m, and 1.3 m from the pile center after construction in April decreased by 1.9, 1.5, and 1.2 °C, respectively, with the refreezing time reduced by 15, 14, and 16 d, respectively.

## 6. Discussion

The discrepancy between the numerical and actual temperature profiles highlights the need to adjust the heat of hydration parameters and the timing of the construction season in the model, as well as the variability between the numerical model and the actual construction operations, in order to better simulate real-world conditions. This insight is particularly important for construction projects in permafrost regions, where thermal management is critical to the structural integrity and service life of pile foundations.

Compared with the ground temperature in the current month, the choice of construction season was more important in the construction of cast-in-place piles; there was a lag in the ground temperature compared to the air temperature. Therefore, excavation operations during construction disrupt the temperature structure of the natural permafrost, with accumulation of ground temperature and air temperature being the dominant influence. To ensure that the permafrost layer remains frozen, it is not recommended to drill holes for construction during the warmer seasons. In particular, the most drastic temperature change occurred from 1 d to 2 d, which was because the heat flow was proportional to the temperature gradient, and the large difference between the atmospheric and soil temperature led to increase in the heat transfer rate. Therefore, conducting drilling and cast-in-place pile construction during cold seasons (spring and autumn) helps maintain the stability of the permafrost around the piles. This conclusion has significant practical implications for construction in permafrost regions. For example, these findings can guide construction managers in optimizing construction schedules; temperature monitoring systems can be deployed to monitor ground and air temperatures in real-time and construction plans can be adjusted based on the temperature data.

## 7. Conclusions

In this paper, the temperature condition and refreezing process of cast-in-place pile and soil around the pile in a permafrost area have been elucidated through data monitoring and COMSOL Multiphysics numerical simulation. The study examines the impacts of the construction season on the boundary conditions of the pile wall, as well as the molding temperature on the temperature field of permafrost under varying seasonal construction conditions, which provides some theoretical guidance for the future design and construction of bridge foundations in permafrost territories. The main conclusions are as follows:

(1) According to the analysis of the temperature monitoring data of the pile in the test site, the internal temperature variation trend can be divided into three stages: a rapid rise phase (0∼2 d), a rapid decline phase (2∼10 d), and a slow decline and stabilization phase (10∼90 d).

(2) COMSOL Multiphysics is used to simulate the distribution of the soil temperature field and the variation law of the temperature field with space, and it is found that the variation in soil temperature with time is similar in different depth ranges. The closer the radial distance from the pile center, the more rapid is the increase in temperature and the higher the MTR. The occurrence of the MTR shows a lagging effect with increase in distance from the pile center. The further the distance from the pile center, the more gradual is the temperature change rate decrease, and the influence is less. The influence range of the molding temperature and the hydration heat is about 1∼2 times the pile diameter, and the influence range is less than 1.5 m in the depth direction.

(3) The accumulation of ground temperature and air temperature are two important factors affecting the temperature changes of the hole’s walls after drilling. Compared to the atmospheric temperature, there is a lag in the temperature of the permafrost, and the significant difference between the two leads to an increased rate of heat exchange. Conducting drilling operations in the cold seasons, such as spring and autumn, is conducive to maintaining the stability of permafrost on the hole side.

(4) The choice of construction season during the construction of cast-in-place piles should be emphasized. For cast-in-place piles, drilling and construction operations in spring (March, April, and May), both the MTR and the refreezing time of frozen soil are the smallest, followed by autumn (September, October, and November), with summer (June, July, and August) being the least favorable.

In future work, we plan to apply the numerical model to more field data to further verify and optimize it. Furthermore, although it is recommended to carry out construction in cold seasons, as it is conducive to reducing the disturbance of the permafrost around the pile, can the concrete strength guarantee the long-term performance and stability of the structure at low temperatures? Therefore, it is of great significance to further explore the formation and development mechanism of concrete strength under low-temperature conditions.

## Figures and Tables

**Table 1:** Geotechnical geology of bridge foundation.

Lithology	Legend	Strata Thickness/m	Description
Fine sand	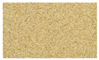	4.4∼4.6	Yellowish brown, moderately dense, moist; dominated by quartz and feldspar with pure sand.
Gravel	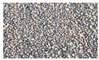	3.0	Grayish-white, slightly moist, medium dense to dense, with a general particle size of 2∼10 mm, and about 10% of the fill is clay.
Boulder	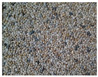	3.8∼4.0	Grayish-brown, moderately dense, slightly moist, with a general particle size of 10∼20 mm, and filled with a large amount of cobblestones.
Fully weathered mudstone	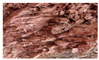	11.4∼12.0	Reddish-brown, clayey structure, primarily composed of clayey soil, approximately 3∼15 cm in length.

**Table 2:** Prototype pile design parameters.

Number	Diameter/m	Design Length/m	Actual Length/m	Piling Method
No.0	1.5	20	21	Cast-in-place pile

**Table 3:** Physical and thermodynamic parameters of modeled soil layers.

Physical Parameters	Depth /m	*w* %	$\rho_{d}$ /(kg/m^3^)	$\lambda_{f}/\left(W\cdot m^{-1}\cdot K^{-1}\right)$	$\lambda_{u}/\left(W\cdot m^{-1}\cdot K^{-1}\right)$	$C_{f}/\left(J\cdot kg^{-1}\cdot K^{-1}\right)$	$C_{u}/\left(J\cdot kg^{-1}\cdot K^{-1}\right)$
Fine sand	4.4	27	1400	3.52	3.78	1810	2150
Gravel	3.0	18	1800	2.98	2.6	2380	2765
Boulder	4.0	10	1600	3.05	2.83	2857	3094
Mustone	11.4∼25	4	2500	2.70	1.98	2290	2290
Concrete	/	0	2300	2.98	2.98	2200	2200

**Table 4:** Comparison of monitoring data with COMSOL calculations.

Time/d	Type	Temperature/°C					MAE/°C
		1 m	4 m	8 m	14 m	18 m	
2	M	29.7	26.0	25.5	25.9	24.8	1.0
	C	27.6	27.1	26.4	25.4	25.2	
15	M	7.0	6.5	10.0	9.4	7.1	2.1
	C	9.0	8.1	11.3	5.6	5.0	
30	M	3.9	3.2	6.2	6.0	4.6	1.7
	C	3.4	4.7	7.2	2.9	2.1	
90	M	2.3	2.0	3.9	3.7	2.9	2.9
	C	−3.7	1.1	1.5	0.8	0.4	

**Table 5:** Effect of construction seasons on refreezing of soil around piles.

	MTR/°C				Refreezing Time/d		
Construction Seasons	0.75 m from Pile Center	1.0 m from Pile Center	1.3 m from Pile Center		0.75 m from Pile Center	1.0 m from Pile Center	1.3 m from Pile Center
April	9.2	4.3	1.5		80	75	62
August	16.6	9.4	5.3		107	100	86
October	11.1	5.8	2.7		95	89	78

## Data Availability

All data to support the results of this study are included in the article.

## References

[1] Subcommittee P. (1988). Glossary of Permafrost and Related Ground-Ice Terms.

[2] Sha A., Ma B., Wang H., Hu L., Mao X., Zhi X., Chen H., Liu Y., Ma F., Liu Z. (2022). Highway constructions on the Qinghai-Tibet Plateau: Challenge, research and practice. J. Road Eng..

[3] Qin T., Xun Q. (2021). Numerical simulation of perturbation of frozen soil temperature field by bored piles. Subgrade Eng..

[4] Wang H., Zhang W., Zhang Y., Xu J. (2022). A bibliometric review on stability and reinforcement of special soil subgrade based on CiteSpace. J. Traffic Transp. Eng. (Engl. Ed.).

[5] Chen K., Yu Q., Guo L., Luo X., Chen J. (2020). Analysis of pile-soil heat transfer process based on field test in permafrost regions. Chin. J. Rock Mech. Eng..

[6] Hou X., Chen J., Yang B., Wang J., Dong T., Rui P., Mei Q. (2022). Monitoring and simulation of the thermal behavior of cast-in-place pile group foundations in permafrost regions. Cold Reg. Sci. Technol..

[7] Chen D., Shao G., Li J. (2017). Experimental analysis on the thermal influence of Cast-in-place Pile’s casting temperature and hydration heat on its surrounding frozen soil. Highway.

[8] Uim F., Coussy O. (1995). Modeling of thermochemomechanical couplings of concrete at early ages. J. Eng. Mech..

[9] Wang Y., Zhao Y., Mao X., Yin S. (2023). Impact of climate change on performance of permafrost highway subgrade reinforced by concrete piles. Future Transp..

[10] Han S., Liu Y., Lyu Y., Liu J., Zhang N. (2023). Numerical simulation investigation on hydration heat temperature and early cracking risk of concrete box girder in cold regions. J. Traffic Transp. Eng. (Engl. Ed.).

[11] Hou X., Chen J., Jin H., Rui P., Zhao J., Mei Q. (2020). Thermal characteristics of cast-in-place pile foundations in warm permafrost at Beiluhe on interior Qinghai-Tibet Plateau: Field observations and numerical simulations. Soils Found..

[12] Shang Y., Niu F., Yuan K., Wu L. (2023). Freezeback behavior of a cast-in-place pile foundation surrounded by two-phase closed thermosyphons in permafrost regions. Appl. Therm. Eng..

[13] Chen P., Ji C. (2015). Monitoring and data analysis of pile temperature field in multi-year frozen zone. Low Temp. Archit. Technol..

[14] Fu J., Jiang Y., Peng H., Dong Y., Yuan K. (2016). Early refreezing law of large-diameter cast-in-place piles in permafrost regions. J. Traffic Transp. Eng..

[15] Li J., Sun X. (2019). Influence of hydration heat on temperature distribution field along piles in warm permafrost regions. J. Railw. Sci. Eng..

[16] Yu D., Cheng P., Ji C., Cui Z. (2016). Study of refreezing of bored pile in high latitudes and low elevation patchy permafrost regions. J. Highw. Transp. Res. Dev..

[17] Qiu K., Yu W., Kong X., Han F., Zhao Y. (2024). Investigation on the bearing capacity evolution of building pile foundation during permafrost degradation. Cold Reg. Sci. Technol..

[18] Wei M., Meng Y., Zhu C., Fang J., Kong G. (2019). Field test on concrete hydration effect of bored pile in backfill soil. J. Disaster Prev. Mitig. Eng..

[19] Wang B., Song Y. (2024). Study on the Hydration Heat Effect and Pipe Cooling System of a Mass Concrete Pile Cap. Buildings.

[20] Sen K., Liu J., Liang R., Wang B. (2022). Study on Thermal Disturbance of Frozen Soil Around Piles by Concrete Hydration Heat. J. Phys..

[21] Zhou Z., Wang D., Zhang L., Ma W. (2015). Determination of large diameter bored pile’s effective length based on Mindlin’s solution. J. Traffic Transp. Eng. (Engl. Ed.).

[22] Freitas J., Cuong P., Faria R., Azenha M. (2013). Modelling of cement hydration in concrete structures with hybrid finite elements. Finite Elem. Anal. Des..

[23] Nielsen C.V. (2007). Modeling the Heat Development of Concrete Associated with Cement Hydration.

[24] Shi H., Huang X. (2009). Research progress of hydration heat in cement and concrete. Cem. Technol..

[25] Zhu Z., Ning J., Song S. (2010). Finite-element simulations of a road embankment based on a constitutive model for frozen soil with the incorporation of damage. Cold Reg. Sci. Technol..

[26] Zhang J., Zhang Z., Guo L., Xie C.L., Jin D.D., Zhai J.B. (2023). Influence of different construction seasons on ground temperature refreezing of con-cylindrical foundation in permafrost regions. J. Jilin Univ. (Earth Sci. Ed.).

[27] Bao W., Liu Y., Mao X., Li W., Qin C., Guo Q., Chen R. (2023). Characteristics of subgrade temperature field of gravel road in high altitude permafrost region. J. Traffic Transp. Eng..

[28] Sun Z., Liu J., Hu T., You T., Fang J. (2023). A solar compression refrigeration apparatus to cool permafrost embankment. Appl. Therm. Eng..

[29] Xiao D., Jiang G., Liao D., Hu Y.F., Liu X.F. (2018). Influence of cement-fly ash-gravel pile-supported approach embankment on abutment piles in soft ground. J. Rock Mech. Geotech. Eng..

[30] Zhang Q., Zhang T., Dong Y., Zhang T., Wei Y., Hao R., Zhao N., Du H. (2024). Thermal performance and applied evaluation of the pre-Bored grouting planted nodular pile in warm frozen soil. Appl. Therm. Eng..

[31] You Y., Yu Q., Guo L., Wang X., Hu J., Qian J., Zhang H. (2016). In-situ monitoring the thermal regime of foundation backfill of a power transmission line tower in permafrost regions on the Qinghai-Tibet plateau. Appl. Therm. Eng..

[32] Xie Y., Yu Q., Yuo Y., Zhang Z., Gou T. (2019). The changing process and trend of ground temperature around tower foundations of Qinghai-Tibet power transmission line. Sci. Cold Arid. Reg..

